# Supplementary material for: Network models reveal high-dimensional social inferences in naturalistic settings beyond latent construct models
Source: Commun Psychol. 2025 Jul 7;3:98. doi: 10.1038/s44271-025-00275-w (PMC12234753; doi:10.1038/s44271-025-00275-w)
Supplement: Supplementary file 2 — Supplementary materials [file 44271_2025_275_MOESM2_ESM.pdf]

Supplementary Materials for

Network models reveal high-dimensional social inferences in naturalistic settings beyond latent construct models

Junsong Lu[1*], Chujun Lin[1,2]

[1] Department of Psychology, University of California San Diego, La Jolla, U.S.

[2] Department of Psychology, Columbia University, New York, U.S.

Junsong Lu https://orcid.org/0000-0001-6987-6228

Chujun Lin https://orcid.org/0000-0002-7605-6508

*Corresponding author *E-mail address*: jul140@ucsd.edu

Table of Content

Supplementary Figures 1 – 3

A

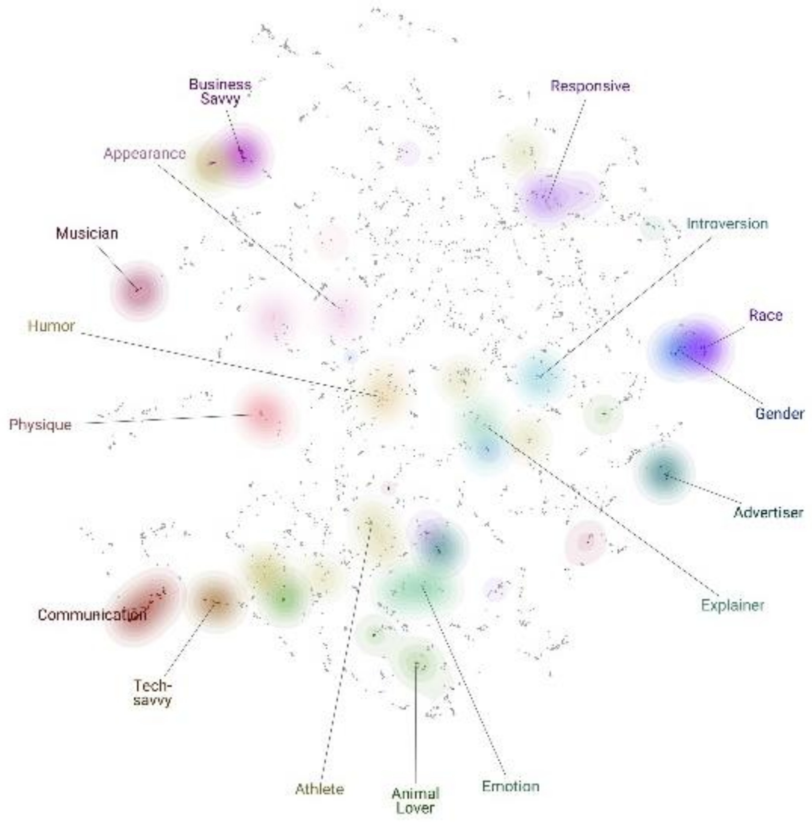
Business Savvy
Responsive
Appearance
Musician
Introversion
Race
Humor
Gender
Physique
Advertiser
Explainer
Communication
Tech-savvy
Athlete
Animal Lover
Emotion

B

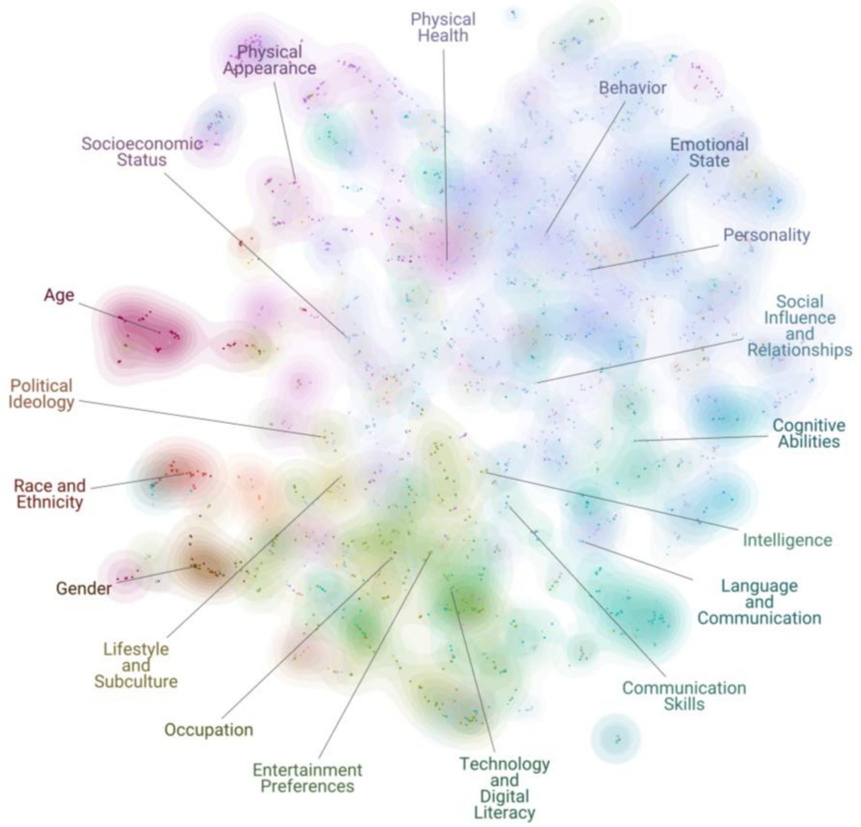
Physical Health
Physical Appearance
Behavior
Socioeconomic Status
Emotional State
Personality
Age
Social Influence and Relationships
Political Ideology
Cognitive Abilities
Race and Ethnicity
Intelligence
Gender
Language and Communication
Lifestyle and Subculture
Communication Skills
Occupation
Entertainment Preferences
Technology and Digital Literacy

Figure S1. UMAP visualization of topic modeling results. (**A**) Results from BERTopic based on the 2,964 unique spontaneous descriptions before lemmatization in Study 1. Dots indicate individual social inferences. Grey dots were identified as noise by the model. Color patches indicate the 16 topic clusters identified by the model. Text labels indicate the interpretation of each topic. (**B**) Results from TopicGPT based on the same set of spontaneous descriptions in Study 1. Color patches indicate the 52 topic clusters identified by the model. Text labels were automatically generated by the model as well, which indicate the interpretation of each topic.

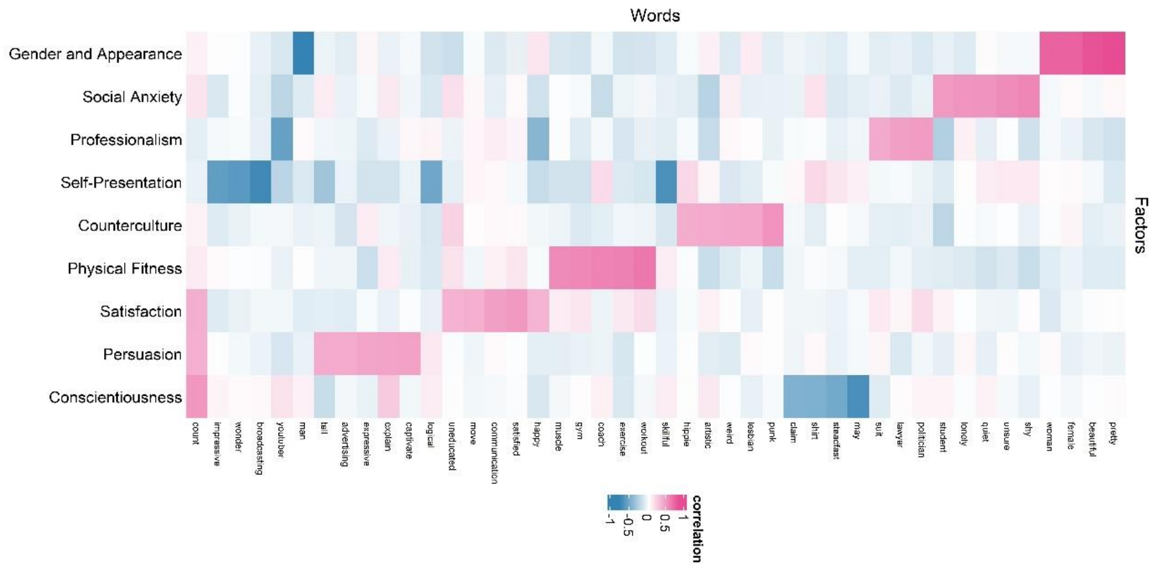

Figure S2. Factor loadings of exploratory factor analysis for Study 2 Asian sample. Exploratory factor analysis on the co-occurrence of the 1,351 social inferences (columns, only the top 5 inferences per dimension are plotted here) freely generated by participants based on 180 naturalistic videos indicated 9 dimensions (rows) optimally underlie the data. Positive loadings are annotated in red; negative loadings in blue. The darker the color is, the greater the absolute value of the factor loading is.

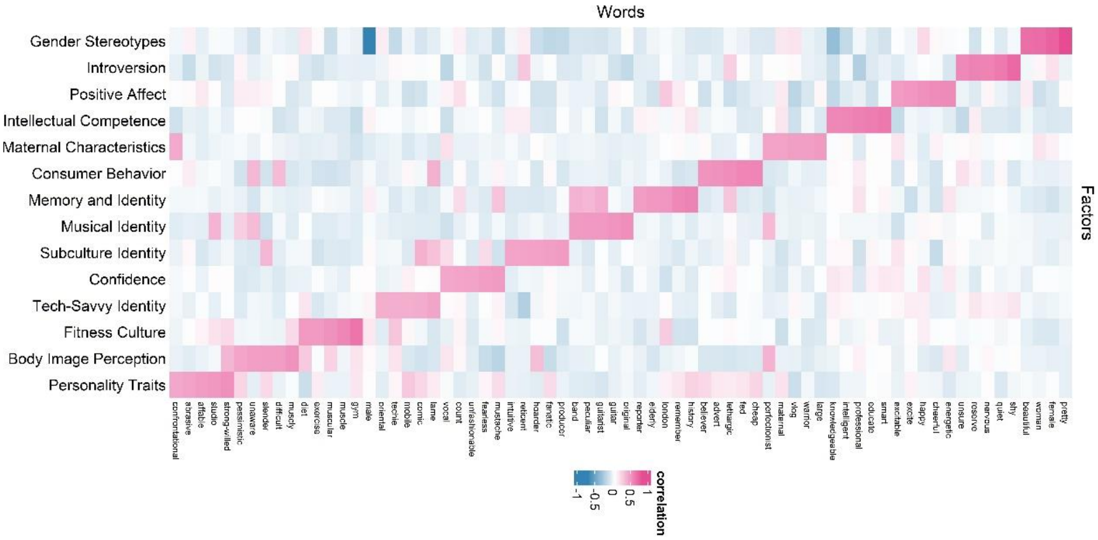

Figure S3. Factor loadings of exploratory factor analysis for Study 2 Europe sample. Exploratory factor analysis on the co-occurrence of the 1,319 social inferences (columns, only the top 5 inferences per dimension are plotted here) freely generated by participants based on 180 naturalistic videos indicated 13 dimensions (rows) optimally underlie the data. Positive loadings are annotated in red; negative loadings in blue. The darker the color is, the greater the absolute value of the factor load is.
